# Use of suppression subtractive hybridization to identify genes regulated by ciliary neurotrophic factor in postnatal retinal explants

**Published:** 2007-02-07

**Authors:** Jérôme Roger, Olivier Goureau, José-Alain Sahel, Xavier Guillonneau

**Affiliations:** 1 INSERM U592, Paris, France; 2 Centre Hospitalier National d'Ophtalmologie des Quinze-Vingts, Paris, France; 3 Université Pierre et Marie Curie-Paris 6 UMR-S592, Paris, F-75012

## Abstract

**Purpose:**

The retinal progenitors are multipotential, and the decision taken by a progenitor to differentiate along a particular path depends on both cell-intrinsic and cell-extrinsic factors. Ciliary neurotrophic factor (CNTF), a member of the interleukin-6 (IL-6) family, added to rat postnatal retinal progenitors inhibits rod photoreceptor cell differentiation, promotes Müller glia genesis and enhances the expression of bipolar neuron markers. We hypothesized that those transcripts regulated during CNTF-influenced retinal differentiation may be involved in the choice of progenitor cell fate. Our aim was to isolate these genes, characterize their expression in the retina, and to subsequently focus on candidates that may promote photoreceptor cell differentiation.

**Methods:**

Retinas were cultured in vitro as explants at postnatal day 0 (P0) in the absence or presence of CNTF for six days. Transcripts regulated by CNTF after six days in vitro (DIV) were selected by subtraction suppressive hybridization (SSH) and cloned as two libraries. The UC6 and DC6 libraries contained those genes upregulated and downregulated, respectively, in the presence of CNTF at 6DIV.

**Results:**

In the first library, UC6, eight clones representing seven different genes were isolated as up-regulated by CNTF. In the DC6 library, 21 clones, representing 17 different genes appeared as down-regulated by CNTF. Genes were classified in six categories, such as protein modification, signal transduction, and regulation of transcription according to the Gene Ontology Annotation.

**Conclusions:**

Among the 24 selected genes, our study revealed 11 genes (two upregulated and nine downregulated) potentially involved in CNTF biological effects.

## Introduction

The generation of the seven major cell types of neural retina follows an evolutionarily conserved temporal sequence, in which retinal progenitors progressively exit the cell cycle and subsequently differentiate in all cell types of an adult retina. Ganglion cells, cone photoreceptors, horizontal cells, and the majority of amacrine cells are born during early histogenesis, whereas bipolar cells, Müller glia, and the majority of rod photoreceptors are born during late histogenesis [1-3]. The retinal progenitors are multipotent, and the decision taken by a progenitor to differentiate along a particular path depends on both cell-intrinsic and cell-extrinsic factors [2-5]. In vitro culture or co-culture experiments have demonstrated that the differentiation of retinal progenitors can be influenced by epigenetic cues such as diffusible factors liberated by differentiated cells or cells undergoing differentiation and exogenously added factors [6-9].

It has been demonstrated that retinal progenitors, and more recently retinal stem cells, retain the ability to differentiate into any retinal cell type, in particular photoreceptors. Thus, these cells offer a promising approach to cure retinal dystrophies and restore visual function in a postmitotic environment such as central nervous system. However, experiments demonstrate that despite the engraftment of these cells in the recipient retina, where they express some retinal markers (in particular photoreceptor markers), they fail to undergo final differentiation and, in the case of photoreceptors, don't exhibit outer segment differentiation [10]. In contrast to stem cells and embryonic retinal precursors, it has been recently reported [11] that rod precursors (derived from P1 to P7 mouse retina) effectively incorporate into the outer nuclear layer of a wild-type or a degenerating retina where they establish functional synapses with cells from the inner nuclear layer (INL). Therefore, to achieve a more complete in vivo differentiation, the mechanism by which photoreceptor differentiation occurs during retinal development needs to be further elucidated. This characterization may be possible by a careful examination of the transcriptome modification during normal development, however, it may be more readily achieved by the analysis of changes in the retinal transcriptome subsequent to the modification of retinal progenitor fate by exogenous cues. Numerous cytokines including fibroblast growth factor (FGF), transforming growth factor β (TGFβ), and taurine, have been described as modifying the development of retina [9]. Among possible cytokines, ciliary neurotrophic factor (CNTF), a member of the IL-6 family was chosen based on its ability to act as a neuroprotective factor on differentiated cells [12] and to affect late retinal neurogenesis (i.e. photoreceptors cells).

CNTF has been described during rodent retinal development, as having an influence on late retinal progenitor fate (in particular a strong inhibition of rod photoreceptor cell differentiation) [13-15], enhancing the expression of bipolar neuronal markers in rat retinal cultures [13,15,16] and promoting Müller glia genesis from the postnatal retinal progenitor pool [17].

In this study, we took advantage of the effect of CNTF on retinal differentiation to identify transcripts whose expression was up- or downregulated during CNTF-induced changes in retinal cell fate, in particular during the inhibition of photoreceptor differentiation. Retinas were cultured as explants at postnatal day 0 (P0) in the absence or presence of CNTF, and transcripts regulated by CNTF after six days were selected by subtraction suppressive hybridization (SSH) and cloned as two libraries: for genes up- or downregulated by CNTF. A differential analysis process revealed eight clones as potential CNTF upregulated genes and 21 as potential CNTF downregulated genes. The aim of our study was to identify genes that promote photoreceptor cell differentiation from progenitor cells; we therefore focused on genes downregulated by CTNF. We explored their expression pattern by real-time PCR on cDNA obtained from retinal explants cultured up to 9 days after addition of either CNTF or leukemia inhibitory factor (LIF). The latter is a CNTF-like cytokine belonging to the IL-6 family, having the same biological effect as CNTF [13,14]. Kinetic expression of selected clones was also performed by real-time PCR during rat retinal development. Their expression in Müller cells and in photoreceptor cells was analyzed by real-time PCR, respectively on cDNA obtained from rat Müller glial cell cultures and in retina from control and dystrophic Royal College of Surgeons rats (RCS).

## Methods

### Retinal explant cultures

Retinal tissues were derived from newborn Sprague-Dawley OFA rats from Charles River (L'Arbresle, France). The methods used to secure animal tissue complied with the ARVO Statement for the Use of Animals in Ophthalmic and Vision Research. Newborn retina were dissected in Hank's Balanced sodium salts (HBSS) without calcium and magnesium (Invitrogen, Cergy-Pontoise, France) and then placed on polycarbonate filter discs (Dutscher S.A., Brumath, France) following directions previously described [18]. Retinal explants were cultured in DMEM/F12 medium (Invitrogen) with 10 mM Hepes pH=7 containing 5% fetal calf serum (FCS) in the absence or in the presence of 10 ng/ml of recombinant CNTF or LIF, purchased from R&D Systems Europe (Lille, France). The medium was changed every three days. After a specified number of days in vitro, explants were fixed for subsequent immunohistochemistry studies or removed for RNA preparation. In this latter case, for each time points, three independent explants were detached from the filters, pooled and washed in PBS without calcium and magnesium before undergoing homogenization in lysis buffer supplied in Nucleospin RNAII kit (Macherey-Nagel, Hoerdt, France).

### Immunostaining

Retinal explant sections were fixed 5 min with 4% paraformaldehyde in PBS before immunostaining with monoclonal antibody against GFAP (Sigma, L'Isle-d'Abeau, France) following previously described procedure [18].

### Müller glial cell cultures

Pure glial cells cultures were prepared from Sprague Dawley rats at postnatal day 10 as described by Goureau et al. [19]. Briefly, eyeballs were incubated in Dulbecco's modified eagle medium (DMEM) containing 0.2% trypsin and 100 U/ml collagenase at 37 °C for 60 min. The globes were dissected to separate the neural retina from the posterior segment and retinas were cut into small fragments and plated in DMEM supplemented with 10% FCS and 2 mM glutamine. After 3-4 days, fragments were removed by extensive rinsing with phosphate buffer saline (PBS), and the remaining flat cell population incubated with fresh DMEM. Primary cell cultures or cells of passages 1-3 were used for experiments.

### RNA isolation and RT-PCR

Total RNA extracted from retinal explants, Müller cell cultures or retina from adult RCS rats were digested by DNaseI (Invitrogen) according to the manufacturer's recommendations. Total RNA were reverse transcribed in presence of oligo-dT using the following protocol. Denaturation of 1 μg of RNA was performed for 5 min, which was then reverse transcribed into cDNA by incubation with 1 μl of deoxyribonucleoside triphosphate solution (dNTP; 10 mM of dATP, dCTP, dGTP, dTTP), 1 μl of oligo-dT (10 μM), 4 μl 5X reaction buffer, 2 μl of 0.1 M Dithiotreitol (DTT), 1 μl of RNasin and x200 U of superscript II (Invitrogen) to a final volume of 20 μl. Samples were incubated for 50 min at 42°C and the enzyme inactivated at 70 °C for 15 min.

### Subtraction suppressive hybridization and cDNA libraries cloning

The polyA RNAs for libraries cloning were obtained from three independent retinal explant cultures. PolyA RNAs were purified from retinal explants total RNA using PolyATtract® mRNA Isolation System I according to the supplied protocol (Promega, France). SSH libraries were generated using the reagents and protocols provided by Clontech (Clontech, Palo Alto, CA); SSH select specific (differentially expressed) transcripts present in a test cDNA population (referred to tester) and absent from a reference (referred to as driver). The first step, two separate samples of tester cDNAs ligated to different 5' adaptor are heat denatured and allowed to anneal with an excess of driver. The concentration of high- and low-abundance sequences is equalized among the single strand cDNA molecules because reannealing is faster for the more abundant molecules due to the second-order kinetics of hybridization. At the same time, single strand cDNA is significantly enriched for differentially expressed sequences. During a second hybridization, the two primary hybridization samples are mixed together without denaturation allowing the single strand cDNA to reassociate and form new type hybrids that can be amplified using primer specific to each 5' adaptor. SSH products are cloned into pCRII-TOPO plasmid (Invitrogen). In the first SSH library, UC6 (upregulated by CNTF after 6DIV), RNA from CNTF-treated explants were used as tester and RNA from control was used as driver. In the second SSH library, DC6 (downregulated by CNTF after 6 days in vitro-6DIV), RNA from control explants were used as driver and RNA from treated explants were used as tester. RT-PCR analysis of the SSH products showed that the level of the housekeeping gene GAPDH decreased more than 100 fold in both (UC6) and (DC6) cDNAs when compared with unsubtracted cDNAs (data not shown), suggesting that the subtraction procedure was effective.

### Differential analysis process and clone selection

For both libraries, a three step analysis was performed. In a first round of screening, 96 clones from each library were randomly collected, grown, and SSH products were amplified by PCR using universal primers. These 96 cDNAs were spotted onto nylon membrane and hybridized with respective subtracted and unsubtracted probes. Clones were selected on the basis of their hybridization pattern according to the manufacturer's instructions. Thereafter, a second round of selection was done using a reverse Northern blot. Briefly, radiolabeled probes were synthesized using cDNA obtained from treated and control explants as template by the ready prime labeling kit accordingly to the manufacturer's instructions (GE Healthcare, Saclay, France). cDNAs selected from the first round were spotted onto new membranes and hybridized with the ^32^P labeled probes. After hybridization and washes, membranes were exposed to Xomat film (Kodak, GE Healthcare Europe). Images were acquired using the GelDoc imaging system (Biorad, France) and analyzed with Quantity One quantification software. For each selected clone, the treated over control intensity-ratio was calculated. Clones with a ratio of 1.5 or higher were selected and considered as upregulated by CNTF, whereas clones with a ratio of 0.5 or below were selected as downregulated by CNTF. Finally, all selected clones were sequenced using the thermosequenase fluorescent kit from GE Healthcare according to the manufacturer's protocol, and run on a Licor sequencer. Sequences were analyzed with BaseImagIR v2.2 software (Licor, ScienceTech, Paris, France) and submitted to BLAST searches of various online databases to characterize genes corresponding to selected SSH products and find identities to human, mouse, and rat sequences. These included the National Center for Biotechnology Information (NCBI; no redundant GenBank, European molecular biology laboratory (EMBL), DNA data bank of Japan (DDBJ), and protein data bank (PDB)), Express Sequence Tag (EST; no redundant GenBank, EMBL, and DDBJ EST divisions), the Ensembl Genome Browser, and the Institute for Genomic Research (TIGR).

### Quantification by real-time PCR

Real-time PCR was performed using an Applied Biosystems 7300 Real-Time PCR System (Applied Biosystems, Foster City, CA). Real-time PCR was performed with Platinum® Quantitative PCR SuperMix-Uracil DNA glycosylase (SuperMix-UDG; Invitrogen) according to the manufacturer's instructions. Reactions were performed in a 20 μl final volume with 1.25 μM primers, 0.4 μl of a glycine conjugate of 5-carboxy-X-rhodamine, succinimidyl ester (ROX), 2 μl of cDNA (1/10) and 10 μl of Platinum® Quantitative PCR SuperMix-UDG containing buffer, MgCl_2_, SYBR Green dye, and dNTP mix (with dUTP instead of dTTP). Primers used are described in Table 1. A typical amplification protocol included an initial incubation of UDG at 50 °C to remove nonspecific amplification that occurred during preparation of plates, a 95 °C initial denaturation step for 2 min followed by 45 cycles with a 95 °C denaturation for 15 s, and an annealing/amplification at 60 °C for 1 min. Detection of the fluorescent product was carried out at the end of the 60 °C extension period. The absence of nonspecific products was confirmed by both the analysis of the melt curves and electrophoresis in 2% agarose gels of the PCR products. All samples were run in triplicate and the fluorescence threshold value (Ct) was determined using the 7300 system software (Applied Biosystems). To adjust the difference of concentration of RNA reverse transcribed, S26, a ribosomal protein, was used as an internal control. Relative quantity was evaluated by ratio of RNA expression of the target gene and S26 [20], expressed in arbitrary unit. Unpaired student's t-tests were used to compare data (Statistica Software; StatSoft, Créteil, France). Values of p<0.05 were considered to be significant.

**Table 1:** Primer sets against rat sequence corresponding to cDNAs identified by subtraction suppressive hybridization.

	**UC6 Library**		**DC6 Library**
**Clone**	**Rat sequence (5'-3')**	**Clone**	**Rat sequence (5'-3')**
9H	F: CTCAATGCCGGCTTCAAAGAG	F5	F: CAGCAGTGACCACCAACTGTC
	R: CGAAGTTCTGCCTGGTAAACG		R: ATCAAACCAAAACCCTTAGGGG
6H	F: GACTCAGAGATGTAAGATCCC	G4	F: TCTTGTATGAGGACTCCGTGC
	R: TGTGCAGAGCTCTCTTCAGAC		R: GGTACAAGAGCCAGCAGCATA
4F	F: ACCCACTAAGCCTCCATTGG	E2	F: CAGAGTCTCCTTTGCCTGAG
	R: GGTCCTGGAATGAAGCCATG		R: GGTATGGATCCAACCCACTG
2G	F: TTCCAGCTTGAAAGCCCAGCC	D2	F: CAAGAGGGCCATCTGTACCA
	R: TGACTCCGGGCTTGTGCGTAA		R: TTCAGGCACGGATGCAGATG
10A	F: GGCGCCTGCGATTAGTGCTACA	A7	F: GGCAGAGGGCGCTTTAATTTC
	R: TGATCCTGCCCAGGCCAAAGA		R: AGTGTCAAGTTCTGCTCCCTG
		F2	F: ATCAATTATGAAAGTTTACTGCC
			R: GCTTTTCTTGGATTCATTTGTC
		D1	F: ATGCCTCCATGTGTAGTGCGG
			R: TTTTGGTTGGTTTGCCTAAAA
		C2	F: GAGATGTACTACTGCTCCAAGTG
			R: GTGGTTTATGCTGGCAAAGAG
		B6	F: ATGGAGATGAAGAAGAAGATTAAC
			R: TGTCACTAAGTTCCAACTTCCG
		B2	F: TGAATTTGGCTCAGGAGCTGC
			R: ACTCAGTGAGTCACAGGAGGA
		G7	F: CTGTGGGCTTACAGAGCCAAA
			R: ATGGCTGCTTCCCTCTATAGT
		C7	F: AGGAAGGAAGAGCCTCGTCGT
			R: AGGGTTGCTGCACTGTTACTA
		A2	F: TCCCCTGAACCTGAATGCCTGG
			R: CTCAGCAGACTGCCCGAGGAA
		S26	F: AAGTTTGTCATTCGGAACATT
			R: GATCGATTCCTGACAACCTTG

Clone denotes the cDNA number in the library, and rat sequence represents forward and reverse primer pairs corresponding to the cDNA. The primers were used to detect gene expression from mRNA extracted from retinal explants or RNA from neonatal rats. DC6 indicates down-regulated by CNTF after 6DIV, UC6 indicates up-regulated by CNTF after 6DIV.

## Results

We generated two rat retina subtracted libraries with the aim of identifying genes whose expression is either up- or downregulated by CNTF. mRNAs were collected from P0 rat retinal explants and cultured with media alone or with CNTF for 6 DIV. To identify genes upregulated by CNTF after 6 days, a library named UC6 was generated using mRNAs from control explants as driver, whereas genes downregulated by CNTF after six days were isolated in a library named DC6 made with CNTF-treated explants mRNAs as driver. The subtractions were assessed by RT-PCR amplification of *Gapdh*, a housekeeping gene for which the expression ex vivo is not influenced by the addition of CNTF. *Gapdh* were not amplified from the two subtracted SSH-cDNAs from 18 to 33 cycles of amplification, whereas it could be amplified with as few as 18 cycles of PCR amplification from nonsubtracted cDNAs (data not shown). The kinetics of *Gapdh* amplification clearly indicated that subtractions were effective.

Following library screening, we isolated eight clones from the UC6 library as potential CNTF upregulated genes and 21 from the DC6 library as potential CNTF downregulated genes. In both libraries several genes were represented by more than one clone indicating the likelihood that the totality of UC6 and DC6 cDNA had been screened. In the UC6 library, the mean size of the cDNA was 421 base pairs and 474 in the DC6 library. This small size was due to the requirement of an enzymatic restriction of cDNA during the library cloning steps for a proper normalization and subtraction of cDNA. Most of the clones were located within the 3' UTR of their corresponding gene. The 29 selected clones were sequenced and submitted to databases to search homology with known rat sequences and to find the human and mouse orthologues. Results are shown in Table 2 and Table 3. All accession numbers refer to NCBI databases.

**Table 2:** Genes isolated by subtractive suppressive hybridization in a UC6 (upregulated by ciliary neurotrophic factor after six days in vitro) library.

**Clone**	**bp**	**Blast result**	**Symbol**	**Accession number**	**Homo**	**Status**
9H	234	Glial fibrillary acidic protein	Gfap	NM_017009	1554	V
6H	692	Pleiotrophin	Ptn	NM_017066	2117	A
4F	270	Oncostatin M receptor	Osmr	AB167522	2972	A
2G	370	Nuclear Receptor coactivator 1	Ncoa1	XM_233944	7859	no
10A	609	Glycyl t-RNA synthase	Gars	BC088347	1547	no
1H	486	RNA binding motif protein	Rbmx	unknown	20494	R
3B	367	Similar to RIKEN cDNA 3930401K13	N/A	BC085931	12252	R

UC6 subtraction suppressive hybridization library was generated using RNA from six days in vitro (6DIV)-ciliary neurotrophic factor treated explants as tester and the RNA from control as driver. cDNAs from this library were screened for differential expression and sequenced. Clone denotes the cDNA number in the library; bp represents the size in base pair of the cDNA; blast result indicates the published sequence identical to the clone sequence; symbol, accession number and Homo refer to the available homologene on-line datas for the published sequence. Status represents the cDNA characterization in this study: A for analyzed, R for rejected, no for gene that didn't exhibit gene expression variation, and V for gene validating the library.

**Table 3:** Genes isolated by subtractive suppressive hybridization in a DC6 (downregulated by ciliary neurotrophic factor after six days in vitro) library.

**Clone**	**bp**	**Blast result**	**Symbol**	**Accession number**	**Homo**	**Status**
GO: apoptosis						
D2	284	Caspase-3	Casp3	NM_012922	37912	V
A7	258	Bcl-2 associated transcription factor	Bclaf	XM_214967	8832	A
GO: Regulation of gene expression						
E2	693	Cone-Rod homeobox protein	Crx	NM_021855	467	V
F2	521	Bromodomain adjacent zinc finger domain 2b	Baz2b	XM_229225	8394	A
G4	574	t-complex associated testis expressed 1 like	Tcte1l	NM_001013228	21304	no
C5	369	Riken cDNA 1500031M22	Dimt1	XM_00106535	0 7047	R
D8	710	C21 ORF66 Isoform D	N/A	unknown	9604	R
GO: Protein modification						
D1	465	Ste-20 related kinase	Spak	AF099990	22739	A
C2	445	Ubiquitin Specific Peptidase 32	Usp32	NM_220798	13066	A
B6	688	Acidic nuclear phosphoprotein 32 family member E	Anp32e	NM_001013200	41519	A
B2	290	Protein tyrosine phosphatase interracting protein	Ptpip51	BC082081	34926	A
G7	103	6 Epm2a interracting protein	Epm2aip	XM_236659	8875	A
D4	240	Riken cDNA 5330408N05 gene	N/A	XM_23000	36420	R
GO: Protein localization						
C7	490	Translocation protein 1	Tloc1	BC099207	2449	A
GO: signal transduction						
A2	501	ADP-ribosylation factor 4	Arf4	NM_024151	55593	A
F11	350	Homolog to TBC1 Domain family mb 15	Tbc1d15	XM_345825	11249	R
GO: Cell Ion Homeostasis						
F5	670	Iron Responsive Element Binding protein 2	Ireb2	NM_022863	11280	no

DC6 subtraction suppressive hybridization library was generated using RNA from control explants as driver and the RNA from six days in vitro (6DIV)-ciliary neurotrophic factor treated explants as tester. Clone represents the cDNA number in the library; bp denotes the size in base pair of the cDNA; blast result indicates the published sequence identical to the clone sequence Symbol, Accession number, and Homologene refer to the available on-line datas for the published sequence. Status represents the cDNA characterization in this study: A for analyzed, R for rejected, no for gene that didn't exhibit gene expression variation, and V for gene validating the library. cDNA were classified accordingly to their gene ontology (GO).

### Identification of genes isolated from UC6 library

Repeated screening steps allowed the isolation of eight potentially upregulated cDNAs in the UC6 library. These eight cDNAs were sequenced and submitted to on-line databases to determine their corresponding genes (Table 2). Among them, two cDNAs (9H and 7E) represented a unique known gene, four cDNAs (2G, 4F, 6H and 10A) were, respectively identical to four different known genes whereas two cDNAs (1H and 3B) had only low identity to known genes or putative genes. 1H, a RNA binding motif protein, and 3B which was similar to a RIKEN cDNA, were not analyzed, since they did not appear to be relevant to our study due to their low homology to known genes. Taken together, five genes were found as potentially upregulated by CNTF at 6DIV. This relatively small number of genes didn't allow a relevant classification using the Gene Ontology Annotation (GOA).

**Figure 1:**
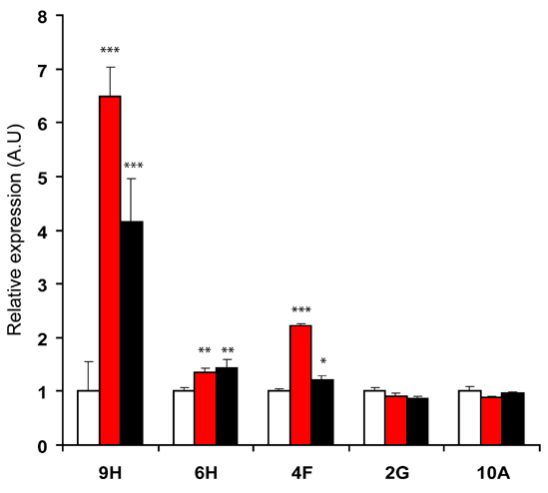
Relative level of UC6 cDNAs expression in retinal explants after six days of cytokine treatment. Retinal explants from P0 rat were cultured for six days in vitro (DIV) with medium alone (white bars) or with 10 ng/ml of either ciliary neurotrophic factor (red bars) or leukemia inhibitory factor (black bars). Retinal explants were harvested after 6DIV and RNA were extracted. cDNA expression corresponding to each clone was analyzed by real-time RT-PCR experiments. Histogram illustrates the mRNA expression corresponding to 9H, 6H, 4F, 2G, and 10A clones normalized by S26. For each gene, all values were compared to mRNA expression of untreated rat retinal explants at 6DIV. Data are expressed as mean±SD from two independent experiments performed in triplicate. (***p<0.001; **p<0.01; *p<0.05).

To ensure that candidate genes were indeed regulated by the CNTF family of cytokines, explants were collected after 6DIV with or without CNTF or LIF in conditions similar to those used for library cloning. Their RNAs were isolated and subjected to reverse transcription followed by real-time PCR amplification. The expression of the five genes was further characterized; results are presented in Figure 1.

**Figure 2:**
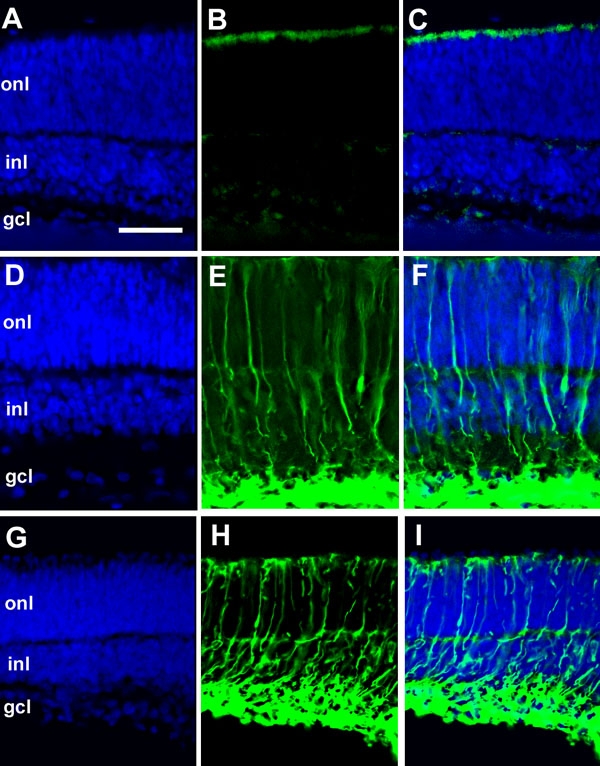
Glial fibrillary acidic protein induction in retinal explants after six days of cytokine treatment. P0 retinal explants were cultured with medium alone (control; **A**-**C**) or supplemented with 10 ng/mL of either ciliary neurotrophic factor (**D**-**F**) or leukemia inhibitory factor (**G**-**I**). After six days in vitro (6DIV) of culture, explants were fixed and stained with anti-glial fibrilary acidic protein (green). Gcl represents ganglion cell layer; inl represents inner nuclear layer; and onl stands for outer nuclear layer. Scale bar equals 40 μm.

9H and 7E cDNAs were identical to glial fibrillary acidic protein (*Gfap*) sequence, which is known to be expressed in activated Müller glial cells [21] and induced by CNTF in adult retina [22,23]. Consistent with CNTF-induced Müller cell differentiation [17], real-time PCR experiments demonstrated that *Gfap* transcripts were induced more than five fold by CNTF in comparison to untreated explants after 6 DIV. In the same conditions, LIF treatment led to a 3.2 fold increase in *Gfap* expression (Figure 1). To confirm the induction of *Gfap* in Müller glial cells by CNTF and LIF, we took flat mounted retinal explants from P0 rats and cultured them in conditions similar to those used for RNA collection. We then analyzed them by immunohistochemistry with a GFAP antibody. In untreated explants (Figure 2B,C), a weak staining was only observed in astrocytes located in the ganglion cell layer, as previously described in retinal sections [22], whereas Müller cells were completely negative. Consistent with real-time PCR data, GFAP staining in the INL was largely increased after the addition of CNTF (Figure 2E,F) or LIF (Figure 2H,I) compared to untreated explants. The lamination of the retina is conserved in the control untreated retina (Figure 2A) after 6DIV as well as after CNTF or LIF treatment for six days (Figure 2D,G).

Clone 6H contained a whole coding sequence and corresponded to a secreted protein named pleiotrophine (PTN), also known as HB-GAM for heparin-binding growth-associated molecule [24]. PTN has been involved in neurogenesis by promotion of neurite outgrowth, nerve cell migration [25,26], and regulation of stem cell proliferation and differentiation [27,28]. Real-time PCR experiments performed at 6DIV demonstrated that CNTF or LIF induced *Ptn* by 35% and 45%, respectively (Figure 1). Due to its biological function, *Ptn* appeared as a candidate gene in mediating CNTF effects on retinal progenitor differentiation. Furthermore as a secreted protein, PTN may be a newly identified exogenous factor capable of modifying the retinal progenitor differentiation as stated in our screen objectives. For these reasons, the characterization of its function during retinal development was given priority. We have recently demonstrated that PTN is involved in the biological effects of CNTF, leading to the inhibition of rod photoreceptor differentiation and has a major role in the increase of bipolar cell differentiation [18].

The 4F clone is a homolog of the oncostatin M (OSM) receptor (*Osmrβ*) sequence. The OSM receptor is a dimeric receptor composed of the gp130 subunit and the OSMRβ subunit, specific to OSM. The other members of the CNTF family (i.e. CNTF or LIF) do not bind to this receptor whereas OSM is capable of binding to either the gp130/OSMRβ or the gp130/LIFRβ complex [29]. Real time PCR experiment performed at 6DIV demonstrated that the expression of this subunit was 2.1 fold higher when CNTF was added to explant culture compared to a control culture whereas LIF treatment led to no more than a 1.2 fold increase in the expression of *Osmrβ* (Figure 1). At 9DIV, under the same conditions, the addition of CNTF or LIF increased by 2.7 fold and 2.8 fold, respectively, the expression of *Osmrβ* suggesting that the difference observed in the induction of *Osmrβ* at 6DIV reflects a delay in the induction by LIF. Since OSM has been described as being able to inhibit rod photoreceptor differentiation [13], the CNTF-induced *Osmrβ* expression in post natal retina may amplify CNTF effects.

The two clones, 10A and 2G, were, respectively homologous to glycyl t-RNA synthase (*Gars*) and Nuclear Receptor coactivator 1 (*Ncoa1*). Real-time PCR failed to demonstrate that these genes were significantly upregulated by CNTF or LIF at 6DIV (Figure 1) even though they passed two differential screen tests.

Surprisingly, despite the quality of the UC6 subtraction, our library failed to identify numerous genes up-regulated by CNTF. This may be due either to the choice of highly stringent conditions for subtraction or also the possibility of overall repression of transcription by CNTF. Previous studies based on analysis of transcriptomic changes in the CNS after CNTF treatment have demonstrated that, as in this study, CNTF mostly induced glial genes [30]. However since we aimed at identifying genes promoting rod photoreceptor differentiation, we next focused our study on genes isolated in the DC6 library.

### Identification of genes isolated from DC6 library

After DC6 library screening, 21 cDNAs were isolated, sequenced, and submitted to on-line databases with the aim of identifying known or predicted genes or EST contigs (Table 3). A few cDNAs were identical to the same gene: D2, F8, C1, C11 corresponding to caspase-3 and A7 and G8 to the Bcl-2-associated transcription factor.

All genes were classified accordingly to their GOA in five functional categories: two cDNAs corresponded to apoptosis (D2, A7), five to regulation of gene expression (E2, F2, G4, C5, D8), six to protein modification (D1, C2, B6, B2, G7, D4), one to protein localization (C7), 2 to signal transduction (A2, F11), and 1 to cell ion homeostasis process (F5). In addition, cDNAs may have had overlapping functions such as B2, which is involved in protein modification and in apoptosis and A7, in regulation of gene expression and regulation of apoptosis (Table 3). Among the 17 different cDNAs, 13 corresponded to identified genes. Of the remaining, one had similarity with a domain homolog of Rab-GAP protein (F11), and three failed to match with any known cDNAs although they showed partial homologies with known domains that enabled their classification using the GOA (C5, D8, and D4). In view of the poor reliability of the data sequences, we decided to exclude these latter four clones from further analyses.

**Figure 3:**
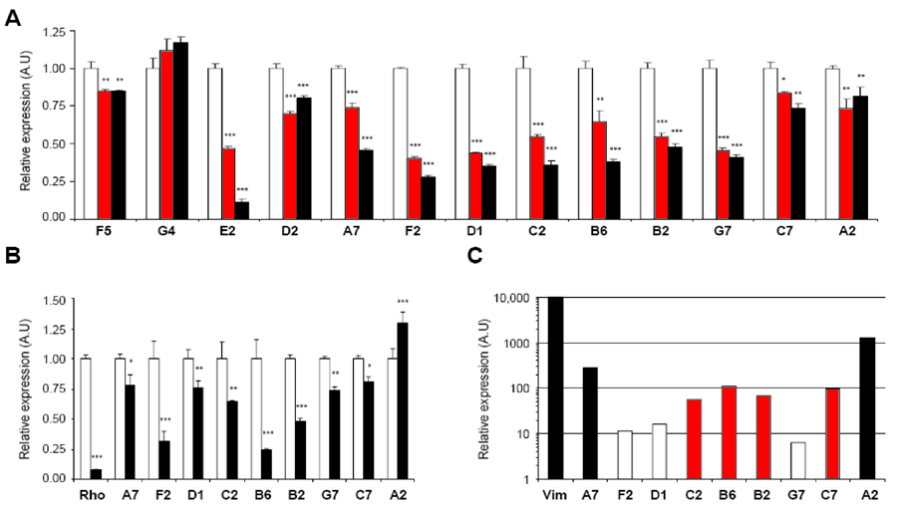
Relative level of DC6 cDNAs expression after six days of cytokine treatment, in RCS rat retina and in Müller glial cell cultures. **A**: Retinal explants from P0 rat were cultured with medium alone (white bars) or with 10 ng/ml of either CNTF (red bars) or LIF (black bars). Retinal explants were harvested after 6DIV and RNA were extracted. cDNA expression corresponding to 13 clones (F5, G4, E2, D2, A7, F2, D1, C2, B6, B2, G7, C7, and A2) isolated from DC6 library was analyzed by real-time RT-PCR experiments. For each gene, all values were compared to mRNA expression of untreated rat retinal explants at 6DIV. Data are expressed as mean±SD from two independent experiments performed in triplicate. (***p<0.001; **p<0.01; *p<0.05) **B**: Retinas from control RCS rat (white bars) and dystrophic RCS rat (black bars) were collected at three months and RNA extracted. cDNA expression corresponding to rhodopsin and to nine clones (A7, F2, D1, C2, B6, B2, G7, C7, and A2) isolated from the DC6 library and differentially expressed upon addition of CNTF or LIF, was analyzed by real-time PCR. For each gene, all values were compared to mRNA expression of control RCS rat retina. Data are expressed as mean±SD from two independent experiments performed in triplicate. (***p<0.001; **p<0.01; *p<0.05). **C**: RNA was extracted from pure retinal Müller cell culture and cDNA expression corresponding to nine clones (A7, F2, D1, C2, B6, B2, G7, C7, and A2) analyzed by real-time PCR. The expression of vimentin (Vim), a glial specific gene, was assessed as a control and arbitrarily fixed at 10,000 u.a. The cDNAs from DC6 library were classified in three groups based on their level of transcription ranged from 6 to 1255 u.a.: Low (white bars, 500 fold less transcripts detected than vimentin transcripts), Intermediate (Int; red bars, 50 fold) and high (black bars, 5 fold).

As for UC6 cDNAs, expression of the 13 remaining cDNAs from DC6 library was first assayed by real-time PCR amplification of RNA from rat retinal explants. The explants were cultured with or without CNTF or LIF for 6 DIV to confirm their down-regulation by these cytokines (Figure 3A). Among the selected cDNAs, 11 reached a minimum inhibition of 25% at day 6 of cytokine treatment (with a mean value of 47% of inhibition) whereas 2 cDNAs were not or were poorly down-regulated by CNTF or LIF after 6DIV. These two clones, F5 (IREB2 for iron responsive element binding protein 2) and G4 (TCTE1L for t-complex associated testis expressed 1 like) were therefore discarded from further expression analysis. Noteworthy, E2 and D2, which corresponded, respectively, to cone rod homeobox protein (CRX), a transcription factor involved in photoreceptor differentiation [31], and to the apoptotic effector, Caspase-3, were downregulated at 6DIV after addition of CNTF or LIF. *Crx* expression was two- and nine fold lower in CNTF- and LIF-treated explants, respectively, compared to control explants, whereas *Caspase-3* expression was decreased by 30% and 21% in CNTF- and LIF-treated explants, respectively, compared to control explants (Figure 3A). The isolation of *Crx*, and *Caspase-3* as downregulated genes is consistent with CNTF-inhibited photoreceptor differentiation [13,15,32] as well as the neuroprotective effect of CNTF on different models of retinal degeneration [12] and therefore demonstrates the quality of the DC6 subtraction.

To redefine the cell type specificity of the nine candidate cDNAs, photoreceptor specific expression was assayed using RNA extracted from a photoreceptorless retina. The expression of the nine cDNAs was quantified in adult dystrophic RCS rat by real time PCR and compared to the expression in an adult nondystrophic control RCS strain. The persistence of cDNA expression in a dystrophic retina reflects expression in the inner part of this tissue. Therefore the ratio of expression of one cDNA in rat retina between the two RCS strains is proportional to its expression in the outer nuclear layer. To confirm the degeneration of photoreceptors in dystrophic RCS rat, we performed the quantification of rhodopsin, a rod-specific protein. In this case, as expected, rhodopsin expression was 12.9 fold lower in the retina of dystrophic animals compared to control RCS rat retina (Figure 3B). Eight cDNAs showed lower expression in dystrophic RCS rat retina than in control RCS rat retina with a mean value of 2 fold. The expression of the cDNA corresponding to A2 was 1.3 fold higher in dystrophic RCS rat than in control RCS rat retina. The level of expression in Müller cells was assayed by real-time PCR using RNA extracted from pure retinal Müller glial cell cultures [19]. The expression of vimentin, a well documented marker of Müller cells [19,33], was arbitrarily fixed at 10,000 u.a. and the expression of the nine cDNAs ranged from 6 to 1255 u.a. (Figure 3C). cDNAs were classified in three groups based on their level of transcription compared to the expression of vimentin: Low, (500 fold less transcript detected than vimentin transcript), including F2, D1 and G7, intermediate (50 fold), including C2, B6, B2, and C7, and high (five fold), including A7 and A2 (Figure 3C).

**Figure 4:**
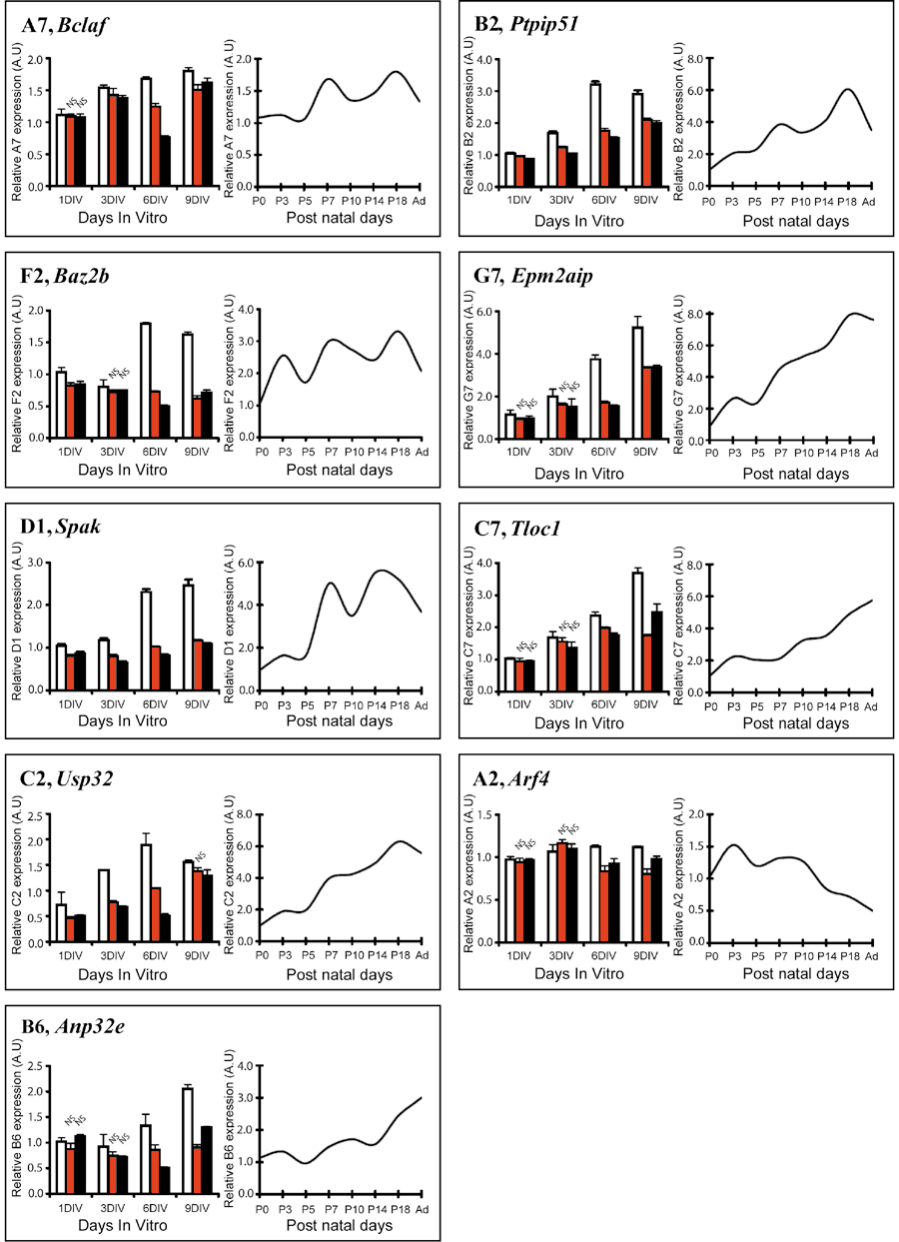
Relative level of differential DC6 cDNA expression after six days of cytokine treatment and during postnatal rat retinal development. Retinal explants from P0 rat were cultured with medium alone (white bars) or with 10 ng/ml of ciliary neurotrophic factor (CNTF; red bars) or leukemia inhibitory factor (LIF; black bars). Retinal explants were harvested after 1, 3, 6, and nine days in vitor (9DIV) and RNA extracted. cDNA expression corresponding to A7, F2, D1, C2, B6, B2, G7, C7, and A2 clones was analyzed by real-time RT-PCR. The histograms illustrate the mRNA expression corresponding to each clone normalized by S26. For each gene, all values were compared to mRNA expression of untreated rat retinal explants at 1DIV. Data are expressed as mean + SD from two independent experiments performed in triplicate. For each time points, values of CNTF- or LIF-treated samples were compared to time-matched control samples. All values were considered as significant (p<0.05) except for those marked NS (Not Significant). Rat retinas were harvested at P0, P3, P5, P7, P10, P14, P18, and adulthood and the RNA extracted. cDNA expression corresponding to A7, F2, D1, C2, B6, B2, G7, C7, and A2 clones was analyzed by real-time RT-PCR. The curves illustrate one representative experiment of the mRNA expression corresponding to each clone normalized by S26. For each gene, all values were compared to mRNA expression at P0.

The kinetics of regulation by CNTF and LIF of the nine selected DC6 cDNAs was therefore analyzed on explants for 9 DIV and during in vivo retinal development. Explants were cultured with or without CNTF or LIF in conditions similar to those used for library. Following varying time-points in culture (1, 3, 6, and 9DIV) explants were collected and their RNAs were isolated and quantified by real-time PCR amplification. Similarly, RNAs were collected from rat retina from P0 to P18 and adulthood and were subjected to reverse transcription followed by real-time PCR amplification. Results are presented in Figure 4. Each DC6 cDNA is described in the following section; cDNAs are listed according to their gene ontology.

### Apoptosis

In addition to Caspase-3, a second gene involved in apoptosis, Bcl-2 associated transcription factor (*Bclaf*), was isolated in the DC6 library (clone A7). *Bclaf*, also named *Btf*, was identified in two separate yeast two-hybrid screens: one using E1B 19K, an anti-apoptotic protein from adenovirus [34] and the second against Emerin, an integral nuclear membrane protein, involved in the Emery-Dreifuss muscular dystrophy [35]. BCLAF is considered a death-promoting transcriptional repressor, depending on its subcellular localization: It represses transcription when localized in the nucleus and acts as a proapoptotic protein when localized in the cytoplasm where it binds to the BCL-2 family members and inhibits their anti-apoptotic activity [34,35].

Real-time PCR experiments on retinal explants confirmed that *Bclaf* expression was downregulated by CNTF (Figure 4, A7). At 6DIV, corresponding to the time chosen for mRNA subtraction, the inhibition of *Bclaf* expression by CNTF or LIF reached a maximum. At this time-point, *Bclaf* expression was inhibited by 26% and 54% after CNTF or LIF treatment, respectively, when compared to control retinal explants (Figure 3A). Analysis of temporal expression of *Bclaf* during postnatal retinal development showed that its expression level remained stable from P0 to adulthood (Figure 4, A7). *Bclaf* expression in dystrophic RCS rat was 1.3 fold lower than in control (Figure 3B). *Bclaf* was estimated to be highly expressed by Müller cells (Figure 3C). Taken together, these results suggested that BCLAF is not photoreceptor specific.

In this context, the CNTF-downregulated expression of *Bclaf* may be one mechanism by which CNTF mediates its neuroprotective effect. Kasof et al. have described BCLAF as a protein that may be involved in the repression of transcription of survival factors [34]. Therefore, the inhibition of expression of *Bclaf* by CNTF may lead to the expression of *Bclaf*-repressed genes (such as Mdm-2 a potential p53 inhibitor) and hence contribute to the neuroprotective effect of CNTF. Alternatively, it is worth noting that BCLAF may alter the cellular localization and bioavailability of BCL2 and thus prevent its anti-apoptotic activity [35]. *Bclaf* down-regulation by CNTF could increase the amount of BCL2 able to bind Bcl-2-associated X protein (BAX) or other pro-apoptotic proteins thus supporting the anti-apoptotic effects of CNTF.

### Regulation of gene expression

In addition to *Crx*, a second cDNA potentially involved in the regulation of transcription was identified. Clone F2 corresponded to bromodomain adjacent to zinc finger domain 2B (*Baz2b*). *Baz2b* belongs to a family of six bromodomain genes involved in chromatin-dependent regulation of transcription [36]. Real-time PCR performed on retinal explants treated with CNTF or LIF demonstrated that following CNTF treatment the expression of *Baz2b* was inhibited by approximately 20% and 10% at 1DIV and 3DIV, respectively, compared to control explant cultures and was inhibited by 60% at 6DIV and 9DIV. LIF treatment achieved a comparable level of inhibition as CNTF at 1DIV and 3DIV, however *Baz2b* expression was inhibited by 72% and 56% at 6DIV and 9DIV compared to control retinal explants (Figure 4, F2). Analysis of *Baz2b* expression during retinal development showed that its expression increased by three fold from P0 to reach a plateau at P7 (Figure 4, F2). The in vitro experiments demonstrated that *Baz2b* expression reached a maximum during the differentiation of late retinal progenitors, suggesting that it could play a role in photoreceptor, bipolar or Müller cell differentiation or maintenance. The Baz2b expression was 3.2 fold lower in dystrophic RCS rat retina than in control RCS rat retina and was poorly expressed in Müller glial cell transcripts (Figure 3B,C). Previous studies have demonstrated that CNTF injection in the mature retina induced a chromatin remodelling in photoreceptor nuclei [37,38] even though Zeiss et al. failed to demonstrate these changes [39]. Furthermore, preliminary studies have demonstrated that BAZ2B could interact with imitation switch (ISWI) [36]. ISWI is involved in chromatin remodeling and in stem cell self-renewal [40]; its activity is dependant upon the formation of the ATP-dependent chromatin assembly and remodelling factor (ACF) complex composed of ISWI and a BAZ family member. Morpholino studies have revealed that ISWI regulates the expression of *Pax6*, *Shh*, *Sox9*, and *Bmp* [41]. Furthermore a dominant negative mutant of ISWI in *Xenopus* revealed profound retinal malformation including aberrant ganglion cell layer formation and a drastic reduction of photoreceptor cell number [41]. We could postulate that CNTF-induced disruption of *Baz2b* expression might inhibit ACF activity, recapitulating the effect of the dominant negative ISWI mutant and therefore participating in the inhibition of photoreceptor differentiation by CNTF.

### Protein modification

Clone D1 corresponded to *stk39*, a Ste20-related kinase (*Spak*) [42], a proline alanine-rich kinase (*Stk39*) belonging to the GCK-VI subfamily of the *Ste* kinase group [43,44]. *Spak* is expressed ubiquitously and has been described as a regulator of the Na-K-Cl cotransporter (NKCC1) in the central nervous system [44,45]. In explants treated with CNTF or LIF, the inhibition of *Stk39* expression peaked at 6 DIV. At this time-point, *Stk39* expression was inhibited by 56% and 65% in retinal explants treated with CNTF or LIF, respectively, compared to control. At 9DIV, CNTF or LIF treatment inhibited the *Stk39* expression by 53% and 56%, respectively. Conversely, *Stk39* expression during rat retinal development was 4 fold higher at P7 than at P0 (Figure 4, D1) and remained constant thereafter. The increase of *Spak* expression following the differentiation of late retinal progenitors during the first post natal week suggests that this protein could be involved in the differentiation of these cells. D1 cDNA expression was 1.3 fold lower in dystrophic RCS retina compared to control RCS retina, suggesting that it is not restricted to photoreceptor cells in retina (Figure 3B). Real-time PCR with cDNA transcripts from Müller cells suggested that D1 was poorly expressed in these cells (Figure 3C). The STE20 family has been reported to be involved in many biological processes including cell cycle, apoptosis, homeostasis, and stress response [43,44]: therefore, SPAK could be involved in the regulation of these processes during retinal progenitor cell differentiation. Consistently, mutations in *Hippo*, a member of the GCK-II subfamily of *Ste20*, have been associated with eye malformations due to an increase in proliferation and a reduction in apoptosis [46].

Clone C2 corresponded to the ubiquitin specific peptidase 32 (*Usp32*), also named *Tre-2*, a protein involved in the deubiquitination pathway. It is described as an oncogene with ability to transform mouse NIH 3T3 cells [47,48] and could be regulated by Calcium/calmodulin complex [49]. *Usp32* expression and function has not been described in the central nervous system. Real-time PCR on retinal explants revealed a maximum decrease of its expression upon addition of CNTF or LIF at 3DIV and 6DIV. *Usp32* expression was inhibited by 45% at 3DIV and 6DIV in CNTF-treated retinal explants compared to control retina and 51% and 73% at 3 and 6DIV, respectively, in LIF-treated retinal explants compared to control (Figure 4, C2). The level of expression increased during retinal development to be maintained at a steady state in adulthood (Figure 4, C2). *Usp32* expression was 1.6 fold lower in dystrophic RCS compared to control RCS retina, while its expression in Müller cell cultures was demonstrated as intermediate (Figure 3B,C). Interestingly, *faf*, another member of the USP family, regulates Drosophila ommatidia differentiation through the deubiquitination of *lqf*, a specific protein involved in the control of photoreceptor differentiation [50,51]. Similarly, USP32 could play a role in vertebrate retinal development by modifying the half-life of key players in eye development.

Clone B6 corresponded to the acidic leucine rich nuclear phosphoprotein 32 family member e (*Anp32e*), also known as *Lanp-l* or *Cpd1* [52-54]. *Anp32e* seems to be expressed in several tissues [55] including neurons where a specific isoform of this protein was involved in synaptogenesis [56]. As shown in Figure 4, B6 expression was inhibited at 6DIV by 35% and 62% in retinal explants treated with CNTF or LIF, respectively, compared to control retinal explants; however no inhibition was observed at 1 and 3 DIV. The maximum inhibition by the CNTF family of cytokines was not reached until 9DIV, with an inhibition of 56% and 37% after CNTF or LIF treatment, respectively, when compared to control retinal explants (Figure 4, B6). The analysis of the expression of *Anp32e* during retinal development indicated that it began to be expressed at P5 and increased until adulthood (Figure 4, B6). *Anp32e* expression was 4.1 fold lower in dystrophic RCS rat retina, compared to control RCS rat retina suggesting that *Anp32e* transcripts are highly expressed in photoreceptor cells (Figure 3B) despite the intermediate level of expression detected in Müller cells (Figure 3C). The absence of inhibition of *Anp32e* expression by CNTF or LIF before 3DIV and the inhibition of expression thereafter may indicate that *Anp32e* is involved in the maturation of the differentiated cells issued from late progenitors rather than directly in progenitor cell differentiation. This hypothesis is supported by the expression of *Anp32e* in photoreceptors and Müller cells as well as its late developmental pattern of expression. Hence, ANP32E may be involved in the final differentiation of photoreceptor cells and in particular in the formation of the synapses in the outer plexiform layer.

Clone B2 corresponded to the protein tyrosine phosphatase interacting protein PTPIP51 and belongs to the family of so-called T-cell protein tyrosine phosphatase-interacting protein (TCPTPIP) [57]. This protein was recently described to be expressed in the retina particularly in the photoreceptor and the ganglion cell layers [57]. *Ptpip51* inhibition by the CNTF family of cytokine peaked at 6DIV. At this time-point, the expression was inhibited upon addition of CNTF or LIF by 45% and 52%, respectively, compared to untreated retinal explants (Figure 4, B2). The level of expression during retinal development showed a biphasic increase of *Ptpip51* transcripts, from P0 to P7 and from P14 to P18 with constant level of expression between P7 and P14. Interestingly, a closed kinetic was observed in retinal explants with an increase of *Ptpip51* expression from 1 to 6DIV and remained constant thereafter (until 9DIV), similar to the in vivo plateau observed between P7 and P14. The expression in retina was 2.1 fold lower in dystrophic RCS rat compared to control RCS rat while the expression was found to be intermediate in Müller cells (Figure 3B,C). A nuclear isoform of TCPTP is described to be involved in the regulation of IL6-mediated signaling pathway through STAT3 dephosphorylation [58]. The potential interaction of PTPIP51 with TCPTP in the retina might be a regulator of the CNTF-activated STAT3, which has been previously reported in the postnatal retina [32,59,60]. In addition, PTPIP51 has recently been described as a mitochondrial protein involved in the induction of apoptosis. In view of this new function, the neuroprotective effect of CNTF might be due to some extent to the CNTF-induced inhibition of *Ptpip51* expression.

Clone G7 corresponded to Epm2a interacting protein (EPM2AIP), which was identified in a yeast two-hybrid screen using Laforin, also named EPM2A, as bait [61]. Mutations in Laforin lead by an unknown mechanism to a rare autosomal recessive disorder named Lafora disease characterized by an impaired glycogen metabolism [62]. This pathology is characterized by epilepsy, myoclonus, and progressive neurological deterioration. EPM2AIP, the first partner of Laforin to be identified, has no significant similarity with any known protein. No inhibition of its expression was observed at 1DIV and 3DIV upon addition of either CNTF or LIF. In contrast, the expression was inhibited by 54% and 36% at 6DIV and 9DIV, respectively, upon addition of CNTF compared to untreated retinal explants (Figure 4, G7). Similar results were obtained with LIF treatment. Expression level during retinal development demonstrated a 9 fold increase from P0 to adulthood (Figure 4, G7). Expression of *Epm2aip* in dystrophic RCS retina was 1.4-fold lower than in control RCS retina and *Epm2aip* was poorly expressed in Müller cells (Figure 3B,C). Hence CNTF is capable of modulating Lafora activity in the eye through regulation of *Epm2aip* expression. An understanding of this regulation might be of use in the development of new approaches to cure this orphan disease and in particular in preventing the formation of Lafora bodies in the retina [63].

### Protein localization

Clone C7 corresponded to the translocation protein 1 gene (*Tloc1*) a homolog of Drosophila *Dtrp1* and yeast *Sec61p*, for which a role in protein translocation across and into the endoplasmic reticulum (ER) membrane has been described [64]. Real-time PCR on retinal explants did not reveal any decrease in *Tloc1* expression upon addition of CNTF or LIF at 1DIV and 3DIV. However, this expression was inhibited by 16% and 53% after 6 and 9DIV, respectively, in CNTF-treated retinal explants compared to control explants. *Tloc1* expression at 6 and 9DIV was inhibited by 26% and 33%, respectively, upon addition of LIF compared to untreated retinal explants (Figure 4, C7). The level of expression increased during retinal development and was maintained at a steady state in adulthood (Figure 4, C7). *Tloc1* expression in dystrophic RCS rat was 0.8 fold lower in dystrophic retina compared to control (Figure 3B) and was classified in the intermediate group for Müller expression (Figure 3C). TLOC1 is classified as a potential nonselective cation channel belonging to the non-selective cation channel-2 (NSCC2) family. Transient-receptor-potential like (TRPL) channel, a Na+, Ca2+ transporter belonging to this family, is implicated with another Transient-receptor-potential (TRP) channel, in the depolarization of the drosophila photoreceptor membrane following light absorption by rhodopsin as well as in the anchoring of the transduction complex in rhabdomere [65]. *Trp/trpl* double mutant drosophila are completely blind [65,66], however constitutive TRP overexpression induces profound drosophila retinal degeneration [67]. Cell death in these models could be due to an increase or a decrease of Ca^2+^ influx. Conversely, in mammals, TLOC1 activity may participate in Ca^2+^-toxicity during retinal degeneration [68]. CNTF-downregulation of *Tloc1* expression may, in this context, facilitate a decrease in intracellular Ca^2+^ imbalance and the subsequent rescue of photoreceptors. Alternatively, together with the clone A2 described below, TLOC1 may be involved in the general trafficking of retinal proteins [64].

### Signal transduction

Clone A2 matched with ADP-ribosylation factor 4 gene (*Arf4*). The ADP-ribosylation factor family is composed of six members distributed in three classes and constitutes one family of the RAS superfamily [69]. *Arf4* is expressed in the retina, where it is involved in the initial steps of the post golgi trafficking of rhodopsin [70]. Mutations that affect the rhodopsin sorting signal interfere with interactions between *Arf4* and rhodopsin, leading to an aberrant trafficking and the initiation of retinal degeneration [70]. Real-time PCR on rat retinal explants treated with or without CNTF or LIF demonstrated that *Arf4* expression was unchanged at 1DIV or 3DIV by CNTF or LIF treatment. After 6DIV, *Arf4* expression was decreased by 26% and 18% in CNTF- and LIF-treated retinal explants, respectively, compared to control retinal explants. At 9DIV, the inhibition of ARF4 was similar to 6DIV, with an inhibition of 28% and 13% (Figure 4; A2). Real-time PCR on retinal cDNA demonstrated that the *Arf4* expression in dystrophic RCS rat was 1.3 fold higher than in control RCS rat (Figure 3A). *Arf4* expression in Müller cells was classified as intermediate (Figure 3B). Real-time PCR experiments failed to show any variation of *Arf4* expression from P0 to P18 nor any decrease after the full differentiation of all retinal cell types. These results strongly suggest that *Arf4* is not exclusively transcribed by photoreceptors. This is in agreement with Deretic et al, who showed expression of ARF4 thought the entire retina [70]. As in other retinal diseases, the original genetic defect may be located in a gene coding for a ubiquitous protein but the outstanding metabolic activity of photoreceptor cells may lead to a photoreceptor-only degeneration [71-73]. In light of our results, *Arf4* is expressed in many cell types throughout retinal development and may be necessary for the proper differentiation of retinal cells. In this context, the downregulation of *Arf4* could disrupt the localization of rhodospin, leading to incomplete differentiation of rod photoreceptors.

## Discussion

CNTF is a potent inhibitor of photoreceptor cell differentiation. Suppressive subtracted libraries were produced and screened with the aim of identifying genes that can mediate the biological activities of CNTF. Our screens have allowed the characterization of several genes regulated by the CNTF family of cytokines, one of which, pleiotrophin has already been established as a regulator of photoreceptor and bipolar cell differentiation [18]. An additional seven genes were characterized as highly photoreceptor specific, such as *Anp32e*, *Baz2b*, or *Ptpip51* or involved in processes related to CNTF activity. The activity of BCLAF and PTPIP51 implicates a role for these proteins in apoptotic events. Furthermore, the observed activity implies a role for USP32, ARF4, and SPAK in controling the activity of key regulators of retinal development by modifying their ubiquitination, localization, or phosphorylation, respectively. The majority of the selected genes were downregulated by CNTF. Consistent with a role of CNTF in adult neuroprotection, the regulation of apoptosis was the category containing the most cDNA with seven different clones. Hence, the mechanism of action of CNTF during development may share similarity with the adult physiology of this cytokine.

All clones selected in both libraries were regulated in the same manner by CNTF and LIF, suggesting that regulation of the DC6 and UC6 genes share a common regulation through the gp130/LIFRβ complex. Noteworthy, in the case of the DC6 cDNA, six out of the nine cDNAs were regulated to a higher extent by LIF than by CNTF. On the other hand, the induction of *Gfap* expression by LIF was 1.5 fold lower than induction by CNTF, reflecting a potentially lower glial response by LIF. Taken together, LIF may be a better candidate for retinal neuron survival and differentiation since it is able to induce a greater regulation of gene transcription with a less detrimental glial response. These effects may be further enhanced since the abundance of LIFRβ/gp130 receptor necessary for LIF activity versus LIFRβ/gp130/CNTFRα complex required for CNTF activity is in favor of the LIF response.

The correlation of the pattern of UC6 and DC6 cDNA expression between in vitro explants and in vivo retinal development observed in this study imply that the role of the isolated candidates may be achieved ex vivo on retinal explants. We previously investigated pleiotrophin (clone 6H) functions by misexpressing it in post-natal retinal explants, and demonstrated that it both inhibited rod differentiation and increased bipolar cell differentiation and thus participates in CNTF-induced effects [18].

Finally our goal was to isolate new candidates with regulatory effects ranging from 25% to several-fold. In this context, our approach using subtractive libraries has enabled the characterization of many genes that would have been eliminated from a broad analysis of the transcriptome, such as DNA chips, where the cut-off is generally set to 2. Previous studies have revealed many differences between the subtractive and DNA chips approach, not only in the range of regulation but also in the selected candidates [74]. DNA chip experiments on retinal explants treated with LIF or CNTF are being undertaken to complete these results and get a better view of the kinetics of these cytokines using cluster analysis. Both approaches are necessary to achieve a comprehensive analysis of the level of transcriptome variation induced by CNTF or LIF and thus help to characterize the genetic programs controling photoreceptor differentiation and survival.
